# Top-down suppression of negative features applies flexibly contingent on visual search goals

**DOI:** 10.3758/s13414-024-02882-x

**Published:** 2024-04-16

**Authors:** Marlene Forstinger, Ulrich Ansorge

**Affiliations:** 1 Department of Cognition, Emotion, and Methods in Psychology, University of Vienna, Liebiggasse 5, 1010 Vienna, Austria; 2 Cognitive Science Hub, University of Vienna, Vienna, Austria; 3 Research Platform Mediatised Lifeworlds, University of Vienna, Vienna, Austria

**Keywords:** Visual attention, Suppression, Top-down, Contingent capture of visual attention

## Abstract

Visually searching for a frequently changing target is assumed to be guided by flexible working memory representations of specific features necessary to discriminate targets from distractors. Here, we tested if these representations allow selective suppression or always facilitate perception based on search goals. Participants searched for a target (i.e., a horizontal bar) defined by one of two different negative features (e.g., not red vs. not blue; Experiment 1) or a positive (e.g., blue) versus a negative feature (Experiments 2 and 3). A prompt informed participants about the target identity, and search tasks alternated or repeated randomly. We used different peripheral singleton cues presented at the same (valid condition) or a different (invalid condition) position as the target to examine if negative features were suppressed depending on current instructions. In all experiments, cues with negative features elicited slower search times in valid than invalid trials, indicating suppression. Additionally, suppression of negative color cues tended to be selective when participants searched for the target by different negative features but generalized to negative and non-matching cue colors when switching between positive and negative search criteria was required. Nevertheless, when the same color – red – was used in positive and negative search tasks, red cues captured attention or were suppressed depending on whether red was positive or negative (Experiment 3). Our results suggest that working memory representations flexibly trigger suppression or attentional capture contingent on a task-relevant feature’s functional meaning during visual search, but top-down suppression operates at different levels of specificity depending on current task demands.

## Introduction

A well-known paradox is that trying not to think about something ultimately prompts its internal representation (Wegner et al., 1987). One possible explanation for why we often fail at intentional ignoring is that working memory representations could inherently facilitate the visual perception of stimuli matching the representation (for reviews, see Noonan et al., 2018; van Moorselaar & Slagter, 2020). For example, features currently maintained in visual working memory might automatically gain access to limited capacity mechanisms enabling their visual processing and (e.g., spatially) fitting motor responses (Desimone & Duncan, 1995; Woodman et al., 2007).

Since humans usually maintain items in their working or short-term memory that are relevant to a current task, an inherent processing advantage for these items would generally benefit goal-directed behavior. In this sense, working memory is closely related to top-down control of visual attention, as the ability to select goal-relevant over irrelevant information from our environment (Wolfe, 2021). For example, humans could successfully search for a target by a working memory-based representation of the target-defining feature(s). This representation could guide attention towards external stimuli with representation- (or memory-) matching features. As a result, the processing of searched-for features kept in visual working memory is faster and more accurate than the processing of features not currently maintained (Desimone & Duncan, 1995; Wolfe, 2021; Woodman et al., 2007).

However, working memory content might not guide visual attention and, thus, facilitate visual processing per se. Instead, the impact of working memory representations on visual attention could depend on or be malleable through top-down search goals. For instance, features maintained in working memory elicit greater attentional capture if reliably associated with a search target instead of with a distractor (Carlisle & Woodman, 2019; Kiyonaga et al., 2012; Olivers & Eimer, 2011). Furthermore, the processing of visual input corresponding to working-memory content might not always be enhanced, but instead also sometimes suppressed based on an observer’s current search goals (Drisdelle & Eimer, 2023; Kerzel & Huynh Cong, 2022; for reviews, see Carlisle, 2022; Noonan et al., 2018; van Moorselaar & Slagter, 2020).

While multiple studies have shown that consistent distractor features can be actively suppressed as a consequence of learning (Cunningham & Egeth, 2016; Gao & Theeuwes, 2020; Stilwell et al., 2019; Wang & Theeuwes, 2018) or task demands (Forstinger et al., 2022; Forstinger & Ansorge, 2023), evidence on flexible top-down suppression of working memory content is controversial and scarce.

On the one hand, previous studies have suggested that under conditions in which flexibility and, therefore, working memory is required, capture by the relevant feature might be inevitable. Such working memory-based attentional capture might be prompted even if the memorized feature would need to be suppressed (for reviews, see Noonan et al., 2018; van Moorselaar & Slagter, 2020). On the other hand, studies have shown that distractor foreknowledge can improve visual search performance by actively guiding attention away from expected and, in this sense, *matching* distractor features (Arita et al., 2012; Carlisle & Nitka, 2019). Since these observations, there has been growing evidence for top-down suppression (for a review, see Carlisle, 2022), leading to a new understanding of working memory-based attentional control: Visual attention seems to be flexibly guided through the facilitation or suppression of features depending on their functional meaning as to-be-searched-for or to-be-suppressed during visual search.

However, evidence is still limited on whether top-down control can quickly initiate and switch between the proactive suppression and enhancement of specific features based on flexibly changing task demands. This is due to previous studies mainly comparing visual search performance in positive or neutral versus negative tasks that were realized in separate blocks (e.g., Arita et al., 2012; Beck et al., 2018; Beck & Hollingworth, 2015; Kerzel & Huynh Cong, 2022; Zhang et al., 2020; Zhang & Carlisle, 2022). Thus, features could be ignored for long sequences of trials, and, hence, there was no evidence that top-down suppression is flexible and possible immediately following a switch between different attentional control mechanisms of guidance versus suppression.

Along similar lines, some researchers have varied positive and negative instructions within blocks, but they have not found any evidence for flexible and selective top-down proactive suppression (de Vries et al., 2019; Reeder et al., 2018). Instead, their findings suggested that when participants receive trial-by-trial instructions on target or distractor features, distractor features trigger suppression in general. This feature-unspecific gating mechanism seems to reduce the likelihood of attentional capture by any distractor, which would be different from the feature-specific top-down and proactive attentional guidance by target-defining (hence, called *positive*) features.

Furthermore, most previous studies instructed participants with distractor features that were not necessary to search for the target. Specifically, the suppression of the distractor features was a non-obligatory “add-on” to facilitate target search further. For example, several previous studies investigated whether observers could use a color cue to guide their attention away from non-target items (e.g., Addleman & Störmer, 2022; Arita et al., 2012; Beck et al., 2018; Carlisle & Nitka, 2019; Drisdelle & Eimer, 2023; Zhang et al., 2020). In these studies, color was not necessary for visual search because the target was defined by a specific circle gap position. Therefore, knowing the color of the target or distractors reduced the number of potential target stimuli and, hence, could speed up visual search, but this color knowledge was not needed for correct target identification (Addleman & Störmer, 2022; Reeder et al., 2018). Instead, participants could have relied on the gap position only.

Although evidence suggests that target-defining positive features guide visual attention even when they are not necessary for target search and an alternative easier strategy would be applicable (e.g., Kerzel & Huynh Cong, 2022), this might not be the case for to-be-suppressed features. This assumption is based on evidence indicating that suppression is mainly restricted to difficult search tasks (Conci et al., 2019) or when using a negative template to suppress a feature is mandatory due to task demands (Kerzel & Huynh Cong, 2022).

Indeed, it makes sense that instructing participants to ignore a helpful but non-obligatory feature may not receive the same priority as processing of an obligatory feature and may, thus, be handled differently (e.g., reactively) to processing of a task-relevant feature that must be used as a search criterion.^1^ Participants may sometimes not even use such non-obligatory features at all (Arita et al., 2012, with set sizes of four; Beck et al., 2018; Conci et al., 2019; Kerzel & Huynh Cong, 2022). Therefore, in the current study, to test the possibility of flexible top-down, feature-specific suppression more exhaustively, we only used task-relevant to-be-suppressed (hence, called *negative*) features to ensure their priority in the attention-guiding representations during target search (Wolfe, 2021).

Taken together, it is still unclear if or how different attentional control mechanisms – for proactive search versus for proactive suppression – operate independently where swift and unforeseeable, and thus, flexible switches between these intentions are necessary. However, answering the question of whether the flexibility of attentional control settings for search versus suppression is possible, is important because it is related to the question of where in memory attentional control of proactive suppression is implemented. When a control setting to search for or to suppress a feature varies blockwise, as in most past research, the respective type of control can be learned and offloaded to long-term memory (cf. Carlisle et al., 2011). In contrast, when search criteria vary unpredictably from trial to trial, working memory is likely responsible for the corresponding proactive top-down control (Kerzel & Witzel, 2019).

Furthermore, the question is also interesting from the perspective of everyday human behavior. For example, if you are dressing for the opera and searching for a jacket that should not be white, “not white” would be a useful negative search criterion, and suppression of the color white would aid in finding a suitable jacket. However, if you then want to pick a white scarf to contrast with the darker jacket, it would be more efficient if the search for the scarf were relatively independent of the previous necessity to suppress the same color (here, white). Yet, swiftly switching between intentions could be challenging, even when they relate to different proactive search goals for positive features. This is indicated, for example, by switching costs (Büsel et al., 2019; Kerzel & Huynh Cong, 2022; Kerzel & Witzel, 2019). The challenge is presumably even higher with switches between control mechanisms for different negative features or between search for positive features on the one hand and suppression of (different or the same) negative features on the other hand because suppression seems to be more difficult than searching (Rajsic et al., 2020).

In the current study, we addressed the following two important open questions on the flexibility of top-down control of visual attention. First, we investigated whether the functional meaning of a task-relevant feature as positive or negative is based on working-memory representations that proactively control visual attention in a flexible way. If working-memory representations account for proactive control of search versus suppression, we expected that positive features proactively guide attention while negative features are proactively suppressed, even if participants have to randomly switch between using positive or negative features to search for the target. Second, we investigated if the degree of guidance versus suppression by one and the same feature depends on the currently relevant task set. Under the perspective that working memory explains proactive search for versus proactive suppression of a feature, we expected that a specific feature should guide attention or be suppressed in response to the current trial’s task demands, even if the tasks and, thus, the features’ functional meanings unforeseeably changed or repeated from one trial to the next.

## Experiment 1

As a starting point, we tested whether task-relevant negative features are flexibly and proactively suppressed based on participants’ current search goals. Furthermore, we investigated whether flexible top-down suppression applies selectively to relevant features, similar to attentional guidance by positive features. Participants were instructed to search for a horizontal bar defined by the absence of one of two negative colors (not red or not blue), and each trial began with a prompt informing participants of the current target identity (a non-red horizontal or non-blue horizontal bar), which alternated or repeated randomly from trial to trial, encouraging working memory-based top-down control (Carlisle et al., 2011; Woodman et al., 2013, 2007). Both negative colors were relevant for the target search due to two factors: First, per each trial, the actual target color alternated between three possible colors (e.g., gray, yellow, and cyan, with non-red targets). Since all three colors were present in each target display (in two non-targets and the target), searching for the target by its actual color was not feasible. Second, both the target and the distractor with the negative color (red or blue) were horizontal, making it impossible to search for the target by its orientation alone. Hence, participants had to use the negative color at some point during a trial to find the target.

In the current study, we measured the influence of positive versus negative features used as search criteria on attentional guidance with the help of peripheral salient cues briefly presented before the target display. Studies have shown that the degree of attentional capture by salient stimuli, such as singletons, does not depend on physical salience but on the singleton feature’s match with a currently used top-down and often feature-specific search criterion. This search criterion is assumed to selectively guide attention through otherwise involuntary capture towards locations with stimuli carrying matching features (Folk & Remington, 1998; Folk et al., 1992; for a review, see Büsel et al., 2020). For example, a salient red cue may capture attention if participants search for red targets. However, it is ignored if participants are searching for a green target. Thus, the influence of a singleton cue feature on attentional guidance is assumed to reflect the feature-specific contents of a proactive top-down control setting.^2^

As is typical, across trials, cue and target positions were uncorrelated. As a consequence, in only 25% of trials, were cue and target positions the same (valid trials), while in 75% they differed (invalid trials). Importantly, since our singleton cues did not predict the target position and were equally likely to appear at any of the four possible stimulus positions, there was no incentive for the participants to direct attention to the cues per se. Thus, any influence of the cue on attentional guidance and on search or reaction times is assumed to emerge involuntarily contingent on a participant’s wrongful application of the attentional control settings (or search goals), set up to search for the targets, to the cue.

Depending on this spatial cue-target relation, we measured the influence of different cue features on visual attention using the validity effect, which was calculated by subtracting mean reaction times in valid from invalid trials. As mentioned above, singleton cues with a top-down matching feature usually involuntarily capture attention, thus facilitating target processing in valid compared to invalid conditions, which results in standard validity effects (Folk et al., 1992; for a review, see Büsel et al., 2020). In contrast, singleton cues with negative features have been shown to elicit inverse validity effects, resulting from slower reactions in valid compared to invalid conditions as some of the cue-elicited suppression would spill over to affect processing of the target presented at the cued location under valid conditions (Forstinger et al., 2022; Forstinger & Ansorge, 2023; Kerzel & Huynh Cong, 2022).

In general, a stronger inverse validity effect for cues with a negative feature (e.g., red with non-red targets) than for non-matching cues with a task-irrelevant feature (e.g., red if the negative feature is green and the target is also never red) would indicate feature-specific suppression of the cue’s negative feature, resulting in diminished target processing in valid conditions (Experiments 1 and 3 in Forstinger et al., 2022). Although salient non-matching cues with task-irrelevant features can also lead to inverse validity effects (e.g., Forstinger & Ansorge, 2023), they might not be all contingent on the need to suppress known irrelevant or distracting features (e.g., Lamy et al., 2004; Schoeberl et al., 2018). For example, inverse validity effects of non-matching cues could reflect some degree of active signal suppression – that is, feature-unspecific suppression triggered by potentially distracting (e.g., salient) features (Gaspelin & Luck, 2018). Additionally, they could reflect object-updating costs associated with the change from cue color to target color at the target position (Carmel & Lamy, 2014). If suppression works in a feature-specific way, inverse validity effects could nevertheless be larger for negative than non-matching cues. However, as explained before, suppression might also be triggered indiscriminately by just any distracting feature.

To increase sensitivity for feature-specific or feature-unspecific suppression and to decrease contributions to inverse validity effects through object updating or other forms of visual forward masking by the cue under valid (non-matching and negative cueing) conditions, two measures were taken. First, we consistently used a very short cue-target stimulus onset asynchrony (SOA) of 60 ms. Prior results have shown that object-updating costs are negligible under such short SOA conditions (Carmel & Lamy, 2015). Second, at all potential stimulus positions, we presented white masking disks in-between colored cues and colored stimuli in the target displays, meaning that object-updating costs or forward masking effects would have been the same under all conditions (e.g., under valid matching cue conditions in a positive search task in Experiments 2 and 3, too).

On the one hand, if flexible top-down suppression of negative features applies proactively and based on current feature-specific search goals, we expected stronger inverse validity effects for cues with a currently used negative color compared to a task-irrelevant non-matching color. For instance, red cues should elicit significant inverse validity effects when participants search for non-red horizontal targets but not for non-blue horizontal targets. Similarly, blue cues should only trigger inverse validity effects in the blue-negative search task. Green cues should elicit non-significant or weaker inverse validity effects in both search tasks because green was always a non-matching color. On the other hand, the flexible use of a negative search criterion may not trigger selective suppression but instead increase the threshold for visual processing (de Vries et al., 2019; Reeder et al., 2018). In that case, we might observe similar inverse validity effects across search tasks and negative and non-matching cue conditions regardless of the current task demands or the identity of the to-be-suppressed feature.

### Method

#### Transparency and openness

All data for the current study were collected in 2023, and the raw data of all experiments are publicly available via the Open Science Framework at https://doi.org/10.17605/OSF.IO/8UE3C. We reported how we decided on the sample size per experiment, data exclusions if applicable, and all independent and dependent variables. This study was not preregistered.

#### Participants

Before collecting data, we planned for a sample size of 30 participants for each experiment. However, if the sample size was below 35 and students had already signed up for participation, we did not turn them away. Our sample size was determined based on previous studies with similar experimental designs (Forstinger et al., 2022; Forstinger & Ansorge, 2023; Kerzel & Huynh Cong, 2022; Zhang et al., 2020) that successfully detected significant effects with a sample size of 20 participants. Nevertheless, we decided to increase this commonly used sample size to achieve sufficient statistical power to detect meaningful effects in our study, given the uncertainties surrounding the research question, if top-down sets for suppression can be applied flexibly.

In addition, for each experiment, we used simulations (Forstinger & Ansorge, 2023; Grüner et al., 2021) to estimate the statistical power to detect significant validity effects in both the negative and the positive search tasks. Specifically, we aimed to simultaneously detect an inverse validity effect of −25 ms in the negative search task and a standard validity effect of 25 ms in the positive search task. These effect sizes were considered the minimum theoretically interesting effect sizes for suppression and capture, respectively. The results of these power simulations are reported alongside the reaction time-based validity effects for each experiment.

All participants received course credits for completing the task and were naïve to the experiment’s purpose. They were treated in compliance with the Declaration of Helsinki’s ethical standards and gave written informed consent before the experiment. Furthermore, all participants had normal or corrected-to-normal visual acuity and no red-green deficiency.

Per sample, we tested for error rate outliers using the generalized extreme Studentized deviate (ESD) test (Rosner, 1983). We tested for a maximum of three error rate outliers and reported *p* values and the test statistic *R* for significant test results. The subscript number of the test statistic *R* refers to the number of tests already performed on the sample (e.g., *R*_1_ refers to the test statistic for the first error rate outlier). In Experiment 1, 32 participants (22 females; *Mdn*_Age_ = 21 years, range 19–25 years) took part. No participant was excluded as an error rate outlier based on the result of a generalized ESD test.

#### Apparatus and stimuli

All experiments were conducted in a dimly lit room. Participants used a chin rest to maintain a stable viewing distance of 57 cm and wore hearing protection to reduce background noise. Stimuli were generated and controlled using PsychoPy (Peirce et al., 2019) and displayed on an LCD monitor with a resolution of 1,920 x 1,080 pixels (54.4 x 30.3 cm) and a refresh rate of 100 Hz. The stimuli were displayed against a dark gray background (CIELAB color space: L* = 35, a* = 0, b* = 0). The fixation point, prompts, rings surrounding the target display stimuli, and feedback text were white (L* = 140, a* = 0, b* = 0) and presented at the screen center (except for the rings). The cueing display, masking display, and target display consisted of four stimuli presented with a horizontal and vertical offset of 4° from the screen center.

#### Procedure

In Experiment 1, we used two search tasks (red-negative and blue-negative) that alternated or repeated randomly from trial to trial. Each trial began with a fixation point (700 ms) followed by the prompt (300 ms), both presented at the screen center. The prompt was a square or a diamond (both shapes had a side length of 2°), and the prompt shape informed participants about the target identity (i.e., a non-red horizontal bar or a non-blue horizontal bar) for the current trial. The assignment between prompt shapes (square or diamond) and search tasks (red-negative or blue-negative) remained consistent throughout the experiment but was counterbalanced across participants.

Next, another fixation display (350 ms) was presented, followed by the cueing display (50 ms). The cueing display consisted of four disks with a diameter of 2°. One disk was colored (i.e., the singleton cue), corresponding to the respective cue condition, while the other three non-singletons were gray. In Experiment 1, we used three cue colors: red (L* = 70, a* = 99, b* = 90), blue (L* = 70, a* = 25, b* = −110), and green (L* = 70, a* = −70, b* = 67). The meaning of the red and blue cue colors varied between search tasks: Depending on the search task, red cues had the negative color in the red-negative search task or were non-matching in the blue-negative search task. Similarly, blue cues had the negative color in the blue-negative search task but were non-matching in the red-negative search task. Green cues were equally non-matching in both search tasks since green was a task-irrelevant color. The cue was presented equally often at each of the four possible positions, independently of the current target position (resulting in 25% valid and 75% invalid trials).

After the cueing display, a masking display was presented for 10 ms, consisting of four white disks with a diameter of 3.5°, one per stimulus position. The masking display was followed by the target display (400 ms). In both search tasks, each target display consisted of two horizontal and two vertical bars (0.2° wide and 1.6° long). In the red-negative search task, one of the horizontal bars was always red (i.e., the negative color), while the other three bars were gray (L* = 70, a* = 0, b* = 0), yellow (L* = 70, a* = 0, b* = 73), and cyan (L* = 70, a* = −41, b* = −20). Each color was present per target display, and unforeseeably, two of the three potential target colors defined vertical non-targets, and only one of them defined the horizontal target. Thus, suppression of the negative color was ensured, and searching for the three potential target colors was not an option: The latter would have guided attention away from the target and towards a non-target in two out of three trials. In each target display of the blue-negative search task, this basic structure was the same, but one of the horizontal bars was blue (i.e., the negative color), while the three other stimuli were gray, yellow, and magenta (L* = 70, a* = 105, b* = −81). As in the red-negative search task, each of these three potential target colors was present in each target display, with only one present in the target and the other two present in the vertical non-targets. For each search task, target display stimulus positions and colors were pseudo-randomized so that each stimulus and color was equally likely to be presented at each position. In both search tasks, each bar in the target display was surrounded by a white ring with a diameter of 3.5° with a gap (2.8° wide) at one of four possible positions (top, bottom, right, or left). Each gap position was realized in each target display and pseudo-randomized across trials. Additionally, each gap position occurred approximately equally often at the target position. After the target display, a fixation display was presented until participants responded or until a maximum of 2 s (the timeout limit) had passed. In both search tasks, participants had to report the gap position in the ring surrounding the target using the four arrow keys on a standard computer keyboard. After participants responded or the timeout limit was exceeded, a feedback display (750 ms) was presented to inform participants whether their response was correct, incorrect, or too slow.

**Fig. 1:**
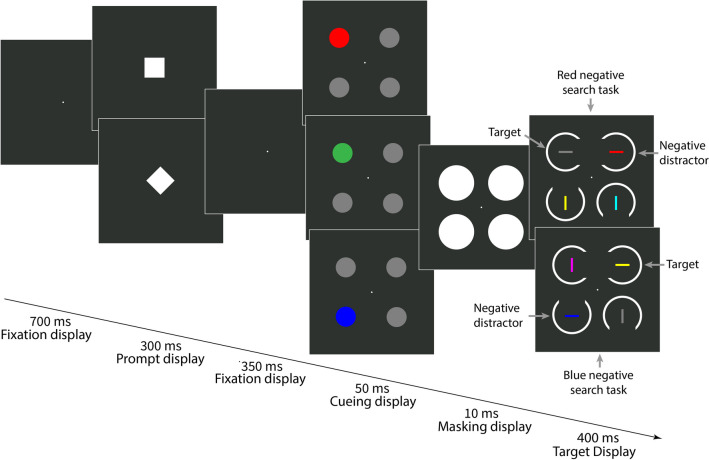
Procedure of Experiment 1. The stimuli are drawn to scale, but the displays are cropped. Each trial started with a prompt (diamond or square) informing participants about the current search task (red-negative vs. blue-negative), which alternated or repeated randomly from trial to trial. Each cue condition (red, blue, and green) is depicted. Singleton cues were presented at the same position as the target (valid condition; 25% of all trials) or at a different position (invalid condition; 75% of all trials). In the red-negative search task (upper target display), the target was a horizontal bar that was not red, and the actual target color changed randomly from trial to trial between gray, cyan, and yellow (here, it was gray). In the blue-negative search task (lower target display), the target was a horizontal bar that was not blue (here, the target was yellow but could also be gray or magenta). In both search tasks, participants had to report the gap position (up, down, left, right) in the ring surrounding the target by pressing the spatially compatible arrow key on the computer keyboard

Before data collection started, participants practiced the task until they achieved 80% accuracy calculated across 20 successive trials. However, a minimum of 25 practice trials was mandatory. Experiment 1 consisted of 1,152 trials, with five self-paced breaks after every 192 trials. The procedure of Experiment 1 is shown in Fig. 1.

### Results

#### Data analysis

We used R (Version 4.2.2, R Core Team, 2020) and the R-packages *broom* (Version 1.0.3; Robinson et al., 2021), *data.table* (Version 1.14.8; Dowle & Srinivasan, 2020), *emmeans* (Version 1.8.4.1; Lenth, 2021), *ggplot2* (Version 3.4.1; Wickham, 2016), *lme4* (Version 1.1.31; Bates et al., 2015), *lmerTest* (Version 3.1.3; Kuznetsova et al., 2017), *MBESS* (Version 4.9.2; Kelley, 2020), *papaja* (Version 0.1.1; Aust & Barth, 2020), and *PMCMRplus* (Version 1.9.6; Pohlert, 2022) for data analysis.

In all experiments, we manipulated cue condition (red vs. green vs. blue in Experiments 1 and 2; red vs. blue in Experiment 3), validity (valid vs. invalid), and search task (red-negative vs. blue-negative in Experiment 1; positive vs. negative in Experiments 2 and 3) as independent variables. Our primary dependent variable was the validity effect, calculated as the difference between mean reaction times in valid and invalid trials (absolute reaction times for each experiment are plotted in the Appendix). We first computed a validity effect separately for each participant, cue condition, and search task, then calculated the mean validity effect across participants for each combination of cue condition and search task.

We also examined the influence of repeated instructions across two consecutive trials on our results. To do this, we compared trials where the search task in the preceding trial (n) was the same (i.e., task-repetition trials) versus different (i.e., task-switching trials) as in the current trial (n+1). Specifically, we recalculated validity effects separately for task-switching and task-repetition trials. Using linear mixed-effects models with task-repetition as an additional two-level fixed factor (task-repetition vs. task-switching), we then compared validity effects based on task-repetition trials to those based on task-switching trials. This allowed us to test whether inverse validity effects in the negative search task depended on task repetitions across at least two successive trials.

In addition to reaction time-based validity effects, we also calculated accuracy-based validity effects as an additional measure of attentional capture by a cue or suppression of a cue. To do this, we first calculated the mean error rate for each experimental condition per participant. We then calculated accuracy-based validity effects for each participant, cue condition, and search task by subtracting the mean accuracy rate in invalid trials from that in valid trials.

We analyzed both reaction time-based and accuracy-based validity effects using linear mixed-effects models (LMMs), with cue condition and search task (and task-repetition) as fixed factors, and random intercepts for each participant. The best-fit model was determined through hierarchical model comparisons (Brown, 2021; Meteyard & Davies, 2020).^3^ For post hoc analyses of validity effects, we used two-sided one-sample *t* tests to assess whether validity effects and validity effect differences between cue conditions within and across search tasks differed significantly from zero.

For all analyses, we adjusted *p* values for multiple validity effect comparisons with the method of Benjamini and Yekutieli (2001) and used a significance level of α = .05. We used Cohen’s *d* as effect size, standardized by the pooled within-subject *SD* and corrected using Hedges’s correction factor (Hedges, 1981), as recommended for small samples (Cumming, 2012).

From the reaction time analysis, we excluded timeouts (reaction times over 2 s; 0.62% of all trials) and wrong trials (18.57% of all trials). In each cue condition of the red-negative search task, 39 of 48 (*SD* = 5) valid trials remained on average, and in the blue-negative search task, 38 of 48 (*SD* = 5) valid trials remained per cue condition. To assess the reliability of our measurements, we calculated intraclass correlations (ICCs) for each combination of cue condition and search task, separately for valid and invalid trials. An ICC2 value of .80 or higher has been suggested as indicating good measurement reliability (Brysbaert, 2019). In Experiment 1, all ICC2 values were above .79, with the lowest value observed for red cues in valid trials of the blue-negative search task.

#### Validity effects in reaction times

**Fig. 2:**
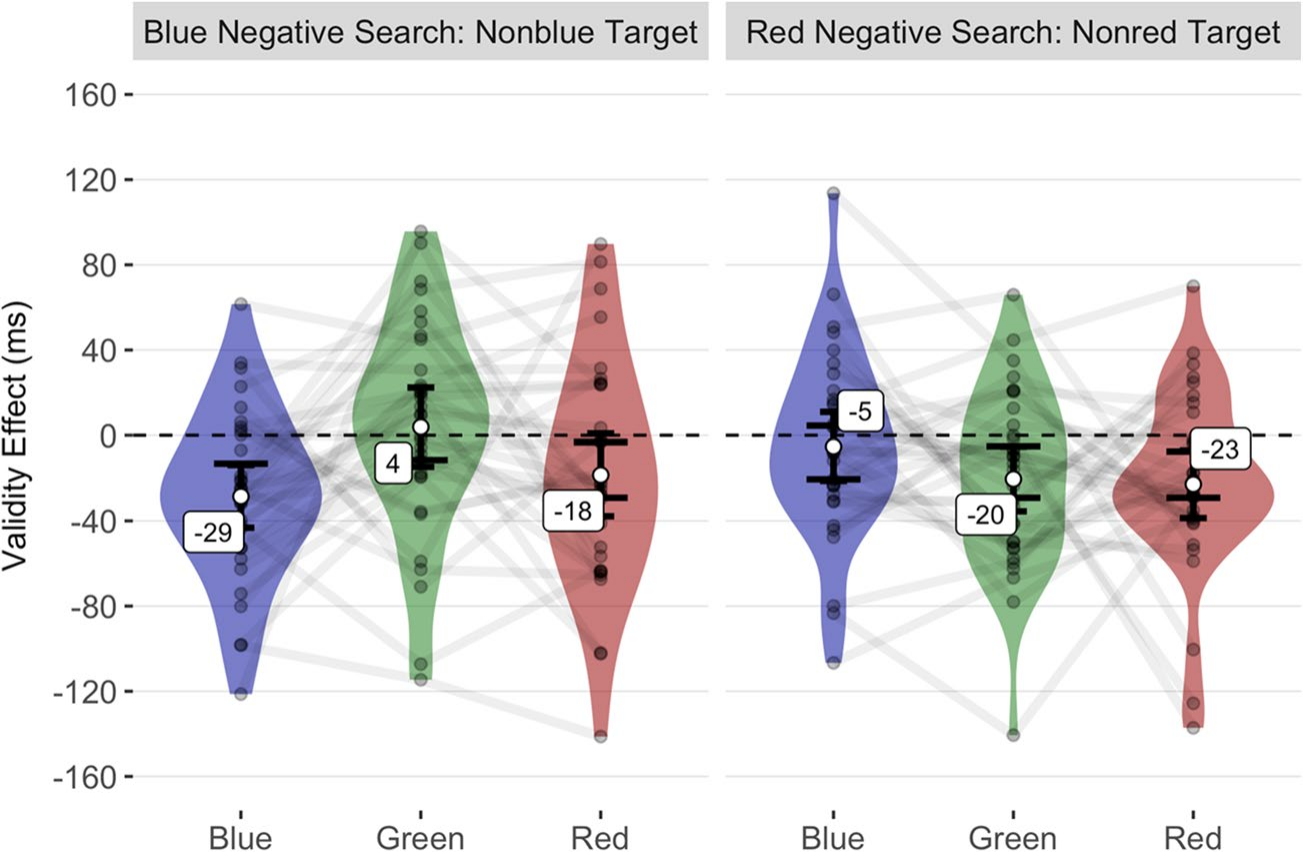
Mean validity **e**ffects of Experiment 1. The mean validity effects are shown on the *y* axis as a function of the cue condition and search task on the *x* axis. The short error bars represent the 95% CIs for the one-sample *t* test against zero ms (black dashed line). The validity effect difference between cue conditions is significant if the long error bars do not overlap. The semitransparent points represent the mean individual validity effects, while the violin plots show their distributions. Lines connect the values of each participant across different cue conditions within search tasks

We found that a model including random intercepts for each participant and an interaction between the fixed factors of cue condition and search task provided a significantly better fit to our data than a model with only the main effects of these fixed factors, χ^2^(2) = 10.08, *p* = .006. Hierarchical model comparisons indicated that adding a main effect of task-repetition to this interaction model did not significantly improve the model fit, χ^2^(1) = 0.10, *p* = .751. Post hoc *t* tests showed that the interaction between cue condition and search task was primarily driven by blue cues producing significant inverse validity effects in the blue-negative search task and non-significant validity effects in the red-negative search task. In contrast, red cues produced non-significant validity effects in the blue-negative search task but significant inverse validity effects in the red-negative search task. Table 1 shows the mean validity effect differences against zero for each combination of cue condition and search task. Table 2 presents the mean validity effect contrasts between conditions. The estimated achieved statistical power to simultaneously detect significant inverse validity effects for red cues in the red-negative search task and non-significant validity effects for red cues in the blue-negative search task was 78%. For blue cues, the estimated achieved statistical power to detect inverse validity effects in the blue-negative search task as significant and non-significant validity effects in the red-negative search task was 79%. Figure 2 illustrates the mean validity effects observed in Experiment 1.

**Table 1:** Mean validity effects in reaction times of Experiment 1

Cue condition	*M*	95% CI	*SD*	df	*p*	*d*_unb_	95% CI
Blue (non-blue Target)	$-$ 29	$-43,-14$	41	31	.006	$-$ 0.68	$-1.08,-0.31$
Blue (non-red Target)	$-$ 5	$-21, 11$	45	31	1	$-$ 0.11	$-0.46, 0.23$
Green (non-blue Target)	4	$-15, 22$	51	31	1	0.07	$-0.27, 0.42$
Green (non-red Target)	$-$ 20	$-36,-5$	42	31	.050	$-$ 0.47	$-0.85,-0.11$
Red (non-blue Target)	$-$ 18	$-38, 1$	54	31	.222	$-$ 0.34	$-0.7, 0.01$
Red (non-red Target)	$-$ 23	$-39,-7$	44	31	.046	$-$ 0.51	$-0.88,-0.15$

*M*, CI (confidence interval) of the mean, and *SD* (standard deviation) in ms. In the column *Cue condition*, the words in brackets indicate the search task (e.g., *non-red Target* means that participants had to search for the non-red horizontal bar as a target)

**Table 2:** Mean validity effect contrasts in reaction times of Experiment 1

Contrast	*M*	95% CI	*SD*	df	*p*	*d*_unb_	95% CI
Non-red Target: Red vs. Blue	$-$ 18	$-42,6$	67	31	.617	$-$ 0.26	$-0.61, 0.09$
Non-red Target: Red vs. Green	$-$ 2	$-26,21$	65	31	1	$-$ 0.04	$-0.38, 0.31$
Non-red Target: Blue vs. Green	15	$-2,33$	49	31	.455	0.3	$-0.05, 0.66$
Non-blue Target: Red vs. Blue	10	$-929$	53	31	1	0.19	$-0.16, 0.54$
Non-blue Target: Red vs. Green	$-$ 22	$-45,0$	63	31	.334	$-$ 0.35	$-0.71, 0$
Non-blue Target: Blue vs. Green	$-$ 32	$-55,-9$	64	31	.099	$-$ 0.5	$-0.87,-0.14$
Red (non-red Target) vs. Red (non-blue Target)	$-$ 4	$-29,20$	68	31	1	$-$ 0.06	$-0.41, 0.28$
Blue (non-red Target) vs. Blue (non-blue Target)	23	$7,40$	46	31	.099	0.49	$0.13, 0.87$
Green (non-red Target) vs. Green (non-blue Target)	$-$ 24	$-44,-4$	56	31	.169	$-$ 0.42	$-0.79,-0.07$

*M*, CI (confidence interval) of the mean, and *SD* (standard deviation) in ms. In the column *Contrast*, the words before the colon or in brackets indicate the search task (e.g., *non-red Target* means that participants had to search for the non-red horizontal bar as a target)

#### Validity effects in accuracies

Based on hierarchical model comparisons, we found no significant effect of cue condition or search task on the accuracy rates of Experiment 1, suggesting that they were similar across experimental conditions (all *p* values ≥ .940).

### Discussion

In Experiment 1, we found that blue cues elicited significant inverse validity effects during the search for non-blue horizontal targets (blue-negative search task) but non-significant validity effects when blue was task-irrelevant because targets were defined as not red (red-negative search task). For red cues, we found significant inverse validity effects when red was the negative color (red-negative search task) but no significant validity effect when red was task-irrelevant (blue-negative search task). Furthermore, these inverse versus non-significant validity effects of red cues in red-negative versus blue-negative search tasks and of blue cues in blue-negative versus red-negative search tasks were not limited to trial-by-trial search-task repetitions. This assumption is based on our finding that adding a main effect of or interactions with the independent variable of trial-by-trial search-task repetition (vs. change) did not improve the fit of the LMM to the data.

However, although red and blue cues elicited non-significant validity effects when these colors were task-irrelevant, such as red in the blue-negative search task, the validity effect contrasts within cue conditions across search tasks were not significant although numerically more pronounced for blue cues, *M* = 23 ms, than red cues, *M* = 4 ms. This finding could suggest a lingering bias toward suppressing the negative color in a search task, when it is no longer relevant. Participants may have sometimes used the wrong search criterion due to frequently switching between negative colors to search for the target.

However, we also observed inverse validity effects for non-matching green cues with non-red targets, which could indicate that participants sometimes suppressed non-matching cues to prevent attentional capture by irrelevant (cue) features, which has been shown in previous studies (Gaspelin et al., 2015, 2017; Gaspelin & Luck, 2018; Lamy et al., 2004). Such suppression of irrelevant cue colors does not result from a corresponding flexible top-down control setting but instead is assumed to reflect a more implicit process similar to selection history (Gaspelin & Luck, 2019). Nevertheless, such suppression could have operated alongside and/or on top of the top-down suppression of negative cue features.

Alternatively, our finding of no significant validity effect contrast within and across cue conditions supports the idea that, to some extent, participants suppressed color in general (see de Vries et al., 2019, and Reeder et al., 2018, for evidence for feature-unspecific color suppression). This assumption seems plausible since color was not a functional positive search criterion that efficiently discriminated the target from non-targets.

Based on the current results, it remains unclear why we observed some degree of feature-unspecificity during the search for a target by alternating negative search criteria, while consistent negative features have been shown to elicit inverse validity effects selectively for negative cues (see Experiments 1 and 3 in Forstinger et al., 2022). Nevertheless, Experiment 1 showed that prioritized working memory representations of task-relevant features do not always facilitate the processing of these features. Instead, while searching for a target by one of different negative features, visual processing of matching (negative cue) features was suppressed, indicating top-down suppression based on current search goals. This conclusion is plausible since we minimized alternative contributions by object-updating costs to inverse validity effects through short cue-target SOAs and color changes between cue and target displays at all potential target locations in all cueing conditions. In addition, given that the cue-target SOA was short and there was, thus, not much time to first attend to the negative cue feature and then suppress it, all before the target was presented, it is likely that the results reflected proactive suppression. In fact, prior research suggested that (more than) 100 ms after distractor presentation and before target (or probe) presentation are necessary for reactive top-down suppression to take effect (Moher & Egeth, 2012; Zhang et al., 2020).

So far, we only focused on flexible top-down suppression. To that end, our participants searched for targets defined by the absence of one of two different negative features. However, in everyday life, attention is often directly guided by positive target features. It is, thus, important to investigate whether participants’ ability to flexibly switch between control processes generalizes to the facilitation of positive features and the suppression of negative features and how this switching between attentional control processes might influence each process (guidance vs. suppression).

Therefore, in Experiment 2, we investigated how flexibly and swiftly top-down control can initiate facilitation versus suppression on a trial-to-trial basis. To that end, search tasks (positive vs. negative) were again randomly intermixed, and a prompt at the beginning of each trial informed participants about the target identity. Addressing this issue becomes particularly interesting in light of Experiment 1. Suppose the task demands for flexibly alternating between unpredictably changing negative search criteria led to the down-weighting of multiple (cue) features in the task-relevant dimension. In that case, switching between suppressing a negative feature and attentional capture by a positive feature within the same dimension might be constrained. In particular, if the negative search criterion (e.g., “not red”) triggered general dimension-based suppression, we might observe inverse validity effects or non-significant validity effects for top-down matching cues (e.g., “blue”) in the positive search task. However, if flexible switching between suppression of negative features and capture by positive features was possible, we expected inverse validity effects in the negative search task and standard validity effects in the positive search task.

## Experiment 2

### Method

**Fig. 3:**
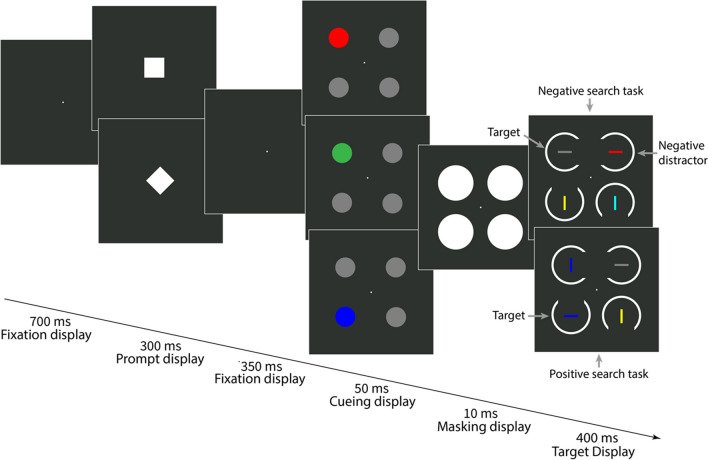
Procedure of Experiment 2. All stimuli are drawn to scale, but the displays are cropped. Each prompt shape (square or diamond) was assigned to a search task (positive vs. negative, counterbalanced across participants). Singleton cues were presented at the same position as the target (valid condition; 25% of all trials) or at a different position (invalid condition; 75% of all trials). In the negative search task (upper target display), the target was a horizontal bar that was not red (here, the target was gray but could also be yellow or cyan). In the positive search task (lower target display), the target was a blue horizontal bar. In both search tasks, participants had to report the gap position in the ring surrounding the target by pressing the spatially compatible arrow key on the computer keyboard

In Experiment 2, we used the same procedure as in Experiment 1, except for a positive search task instead of the blue-negative search task. The negative search task remained identical to the red-negative search task of Experiment 1, where the target was a horizontal bar that was not red. In the positive search task, participants searched for a blue horizontal bar, and, per target display, one horizontal and one vertical bar were blue. Just as in the negative search task, where the target was defined by a combination of two features (a negative color and a positive orientation), the target in the positive search task was also defined by a specific feature conjunction, in this case, of two positive target features (blue and horizontal). In each (blue-)positive search trial, the target display included two other distractor bars, one vertical and one horizontal, that were gray and yellow. Each target display in (red-)negative search trials included the colors gray and yellow along with a third color, cyan, and each of these three colors was alternately present in the horizontal target or in the two vertical non-targets. Thus, per negative search trial, the negative color was again task-relevant because searching for the three potential target colors would have guided attention to a distractor in two out of three trials (if search for three colors would have been possible at all; see Kerzel & Grubert, 2022, for evidence that this is not the case). We used three cue colors (red, blue, and green) as in Experiment 1, but in Experiment 2, blue corresponded to the positive (target) color in the positive search task and a non-matching color in the negative search task. The procedure of Experiment 2 is shown in Fig. 3.

#### Participants

In Experiment 2, 32 participants took part (20 females; *Mdn*_Age_ = 21 years, range 19–32 years). However, two participants were excluded as error rate outliers (outlier_1_ = 58% errors, *R*_1_ = 4.47, *p* < .001; outlier_2_ = 32% errors, *R*_2_ = 3.63, *p* = .001), leaving a final sample size of 30 for data analysis.

### Results

In Experiment 2, we excluded 0.67% of all trials as timeouts, and 9.26% of all trials as wrong trials. In each cue condition of the negative search task, 42 of 48 (*SD* = 4) valid trials remained on average, and in the positive search task, 44 of 48 (*SD* = 3) valid trials remained per cue condition. All ICC2s were above .83, which was found with blue cues in valid trials of the negative search task.

#### Validity effects in reaction times

**Fig. 4:**
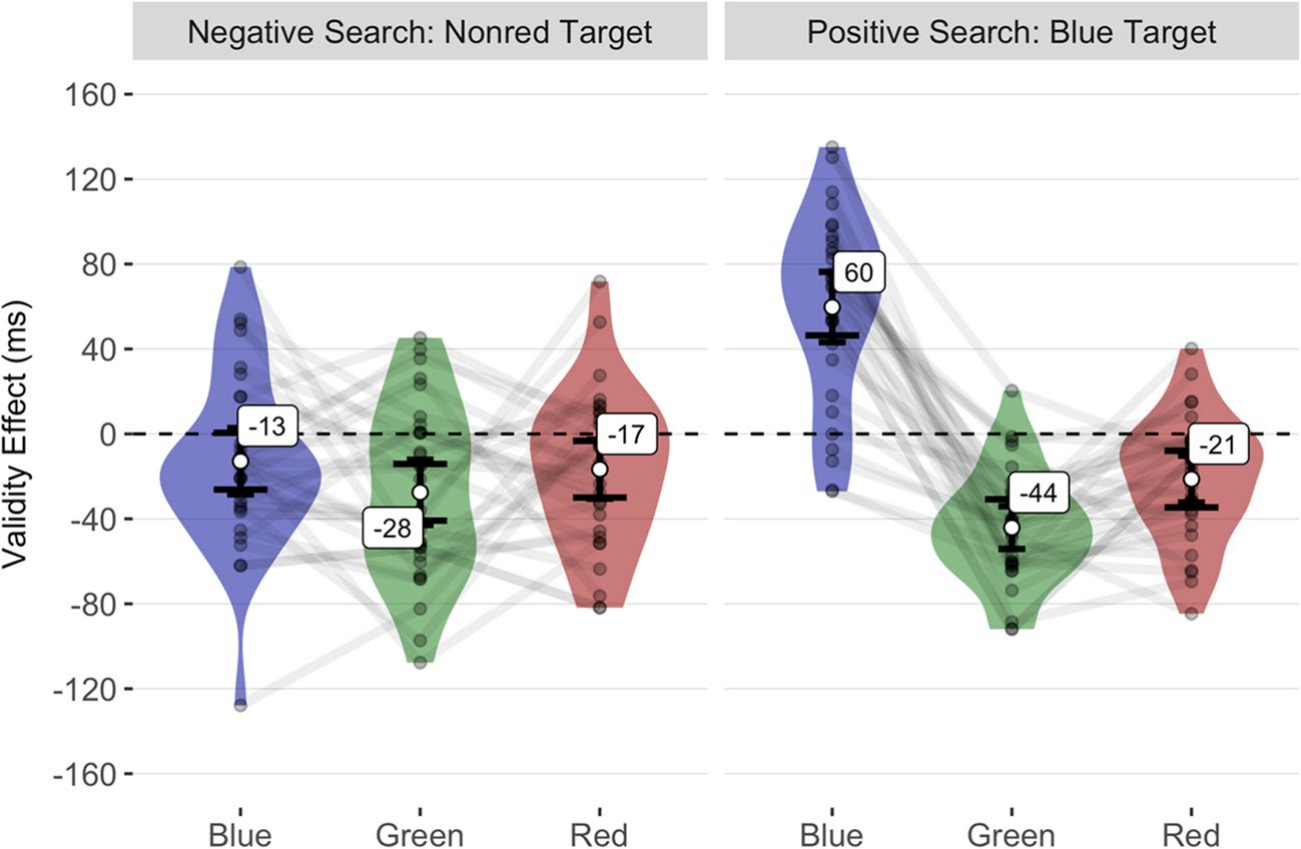
Mean validity effects in reaction times of Experiment 2. The mean validity effects are shown on the *y* axis as a function of the cue condition and search task on the *x* axis. The short error bars represent the 95% CIs for the one-sample *t* test against zero ms (black dashed line). Between cue conditions, the validity effect difference is significant if the long error bars do not overlap. The semitransparent points represent the mean individual validity effects, while the violin plots show their distributions. Lines connect the values of each participant across different cue conditions within search tasks

Hierarchical model comparisons showed that a model including random by-participant intercepts and an interaction between the fixed factors cue condition and search task described our data significantly better than a model including only the main effects of our fixed factors, χ^2^(2) = 48.14, *p* < .001. The significant interaction was based on inverse validity effects for red and green cues across search tasks. In contrast, blue cues triggered significant standard validity effects in the positive search task, but non-significant validity effects in the negative search task. As in Experiment 1, adding a main effect of task-repetition did not significantly improve the model fit, χ^2^(1) = 2.63, *p* = .105. Table 3 presents the mean validity effect differences against zero for each combination of cue condition and search task. Table 4 shows the mean validity effect contrasts between conditions. Our power simulations indicated that the estimated achieved statistical power to simultaneously detect significant inverse validity effects for red cues in the negative search task and significant standard validity effects for blue cues in the positive search task was 94%. The mean validity effects observed in Experiment 2 are shown in Figure 4.

**Table 3:** Mean validity effects in reaction times of Experiment 2

Cue condition	*M*	95% CI	*SD*	Df	*p*	*d*_unb_	95% CI
Blue (non-red Target)	$-$ 13	$-28, 3$	42	29	.253	$-$ 0.3	$-0.67, 0.06$
Blue (blue Target)	60	$43, 76$	44	29	< .001	1.31	$0.84, 1.84$
Red (non-red Target)	$-$ 17	$-31,-3$	37	29	.061	$-$ 0.44	$-0.82,-0.07$
Red (blue Target)	$-$ 21	$-32,-10$	30	29	.002	$-$ 0.7	$-1.12,-0.31$
Green (non-red Target)	$-$ 28	$-43,-12$	41	29	.004	$-$ 0.65	$-1.06,-0.27$
Green (blue Target)	$-$ 44	$-54,-34$	27	29	< .001	$-$ 1.6	$-2.18,-1.08$

*M*, CI (confidence interval) of the mean, and *SD* (standard deviation) in ms. In the column *Cue condition*, the words in brackets indicate the search task (e.g., *non-red Target* means that participants had to search for the non-red horizontal bar as a target)

**Table 4:** Mean validity effect contrasts in reaction times of Experiment 2

Contrast	*M*	95% CI	*SD*	df	*P*	*d*_unb_	95% CI
Non-red Target: Red vs. Blue	$-$ 4	$-24, 17$	55	29	1	$-$ 0.07	$-0.43, 0.29$
Non-red Target: Red vs. Green	11	$-9, 31$	54	29	1	0.19	$-0.16, 0.56$
Non-red Target: Blue vs. Green	15	$-4, 33$	49	29	.471	0.29	$-0.07, 0.66$
Blue Target: Red vs. Blue	$-$ 81	$-100,-62$	50	29	< .001	$-$ 1.59	$-2.18,-1.08$
Blue Target: Red vs. Green	23	$10, 36$	35	29	.009	0.63	$0.25, 1.03$
Blue Target: Blue vs. Green	104	$85, 123$	52	29	< .001	1.96	$1.38, 2.63$
Red (non-red Target) vs. Red (blue Target)	5	$-12, 22$	45	29	1	0.1	$-0.26, 0.46$
Blue (non-red Target) vs. Blue (blue Target)	$-$ 73	$-96,-50$	61	29	< .001	-1.15	$-1.64,-0.71$
Green (non-red Target) vs. Green (blue Target)	17	$-1,35$	49	29	.360	0.33	$-0.03, 0.71$

*M*, CI (confidence interval) of the mean, and *SD* (standard deviation) in ms. In the column *Contrast*, the words before the colon or in brackets indicate the search task (e.g., *blue Target* means that participants had to search for the blue horizontal bar as a target)

#### Validity effects in accuracies

Hierarchical model comparisons showed no significant effect of cue condition or search task on accuracy rates in Experiment 2, indicating that errors were similarly distributed across conditions (all *p* values ≥ .619).

### Discussion

In Experiment 2, we found that blue cues elicited standard validity effects in the positive search task but non-significant validity effects in the negative search task consistent with previous evidence for the flexibility of top-down control in visual search for specific colors (e.g., Grubert & Eimer, 2016; Lien et al., 2010). Furthermore, our results indicate that cues selectively captured attention depending on whether they carried a target-matching feature. In contrast to the selective contingent capture by top-down matching blue cues, we found that red and green cues produced similar inverse validity effects across search tasks, regardless of our instructions.^4^

Some degree of feature-unspecific suppression was also observed in Experiment 1. However, it is interesting that in Experiment 2, inverse validity effects generalized from the negative to the positive search task, where task demands did not require suppression. Theoretically, at least two alternative explanations might account for these findings.

One possibility is that the negative search task encouraged participants to adopt a color-unspecific strategy of suppressing attention to improve search performance across both tasks. Specifically, participants may have established an attentional control setting to search for blue in the positive search task while suppressing all other colors, including negative and non-matching colors. This strategy could have been efficient for overall search performance because, except for blue in the positive search task, color was either an instructed negative feature or a task-irrelevant but potentially distracting non-target feature. This was also the case in our negative search tasks of Experiment 1. Thus, unless we prevent the consistent usage of negative templates for a negative feature (e.g., red) or a consistent non-matching feature (e.g., green), participants may use such templates consistently, even though the task could be performed without them. For example, there was no necessity to suppress the color red in the blue-negative search task of Experiment 1.

Another possibility is that participants used an optimally tuned or relational attentional control setting. This would involve shifting the control setting for the positive color blue to an extreme, shortest wavelength or maybe even toward a wavelength shorter than that of the color blue and at the same time guiding attention away from all longer wavelengths (S. I. Becker, 2010; Navalpakkam & Itti, 2007). This would have been possible since the positive target color was blue, and all distractors had a longer wavelength. Theoretically, participants could have maintained the same relational search setting across all trials, as it would have also worked to guide attention away from the red-negative distractor in the negative search conditions. However, the fact that capture by blue cues was restricted to positive search trials and that the validity effect of blue cues collapsed in negative search trials falsifies this theoretical possibility.

In Experiment 3, we addressed the question of the feature-specificity of the negative search template by testing whether feature-specific inverse validity effects would occur when participants had to use the same feature alternately as a negative or positive search criterion. In this case, consistently suppressing this feature is not an option because it sometimes needs to be used as a positive feature to successfully find the target. In particular, we instructed participants to search for non-red horizontal targets or red horizontal targets. As in Experiments 1 and 2, a prompt presented at the beginning of a trial informed participants about the current target identity. This manipulation should have prevented a lingering bias or strategic decision to suppress salient colors in general because the same color (red) sometimes had to be suppressed and, at other times, to be searched for. As a result, feature-specific inverse validity effects might occur with negative color cues in the negative search task. However, switching between facilitating and suppressing a color based on rapidly changing task demands might be difficult or even impossible. In this case, two outcomes are possible depending on which attentional control setting dominates. First, top-down suppression might be eliminated in the negative search task, indicated by non-significant validity effects for cues with the task-relevant negative color (i.e., red). Alternatively, contingent capture (i.e., standard validity effects) for cues with the target color (i.e., red) might be absent in the positive search task.

## Experiment 3

### Method

**Fig. 5:**
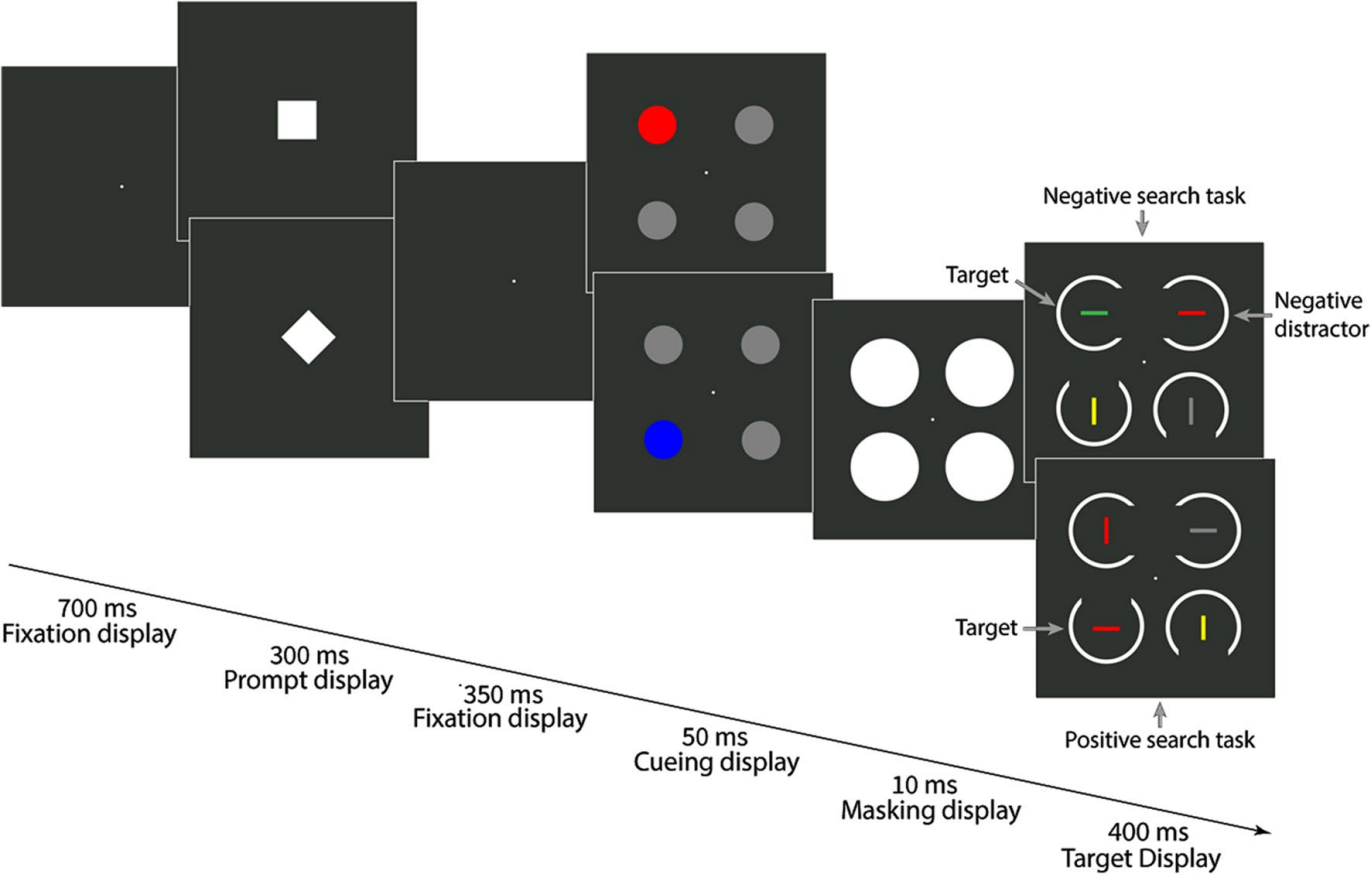
Procedure of Experiment 3. The stimuli are drawn to scale, but the displays are cropped. Each trial began with a prompt (either a diamond or a square) that informed participants about the current search task (negative vs. positive), which alternated or repeated randomly from trial to trial. The assignment of prompt shapes to search tasks was fixed throughout the experiment but counterbalanced across participants. Both cue conditions (red vs. blue) are shown. Cues were presented at the same position as the target (valid condition; 25% of all trials) or at a different position (invalid condition; 75% of all trials). In the negative search task (upper target display), the target was a non-red horizontal bar. In this example, the target was green, but it could also be yellow or gray. In the positive search task (lower target display), the target was conjunctively defined by the presence of two positive features: as a red horizontal bar. In both search tasks, participants had to report the gap position (up, down, left, right) in the ring surrounding the target by pressing the spatially compatible arrow key on the computer keyboard

The procedure of Experiment 3 was similar to that of Experiment 2, with two key adjustments. First, we used the same task-relevant feature for both the negative and positive search tasks. In the negative search task, the target was conjunctively defined by the presence of a positive feature, the bar’s horizontal orientation, and the absence of a negative feature (in this case, red). Again, in the negative search trials, the actual target color changed randomly from trial to trial between gray, green, and yellow, and because the other potential target colors were used for the non-targets, searching for the target color would have guided attention more often to a non-target than to the target. This means that we ensured the suppression of the negative color as an expedient search strategy in the negative search task. In the positive search task, the target was a red horizontal bar. As in Experiment 2, during the positive search, participants had to take both positive features into account, as one distractor had the target color but a different orientation, whereas another distractor had the target orientation but a different color. Specifically, each target display of the positive search task included a red horizontal bar (i.e., the target) and a red vertical bar. Furthermore, the other two bars in the positive search task were yellow and gray, and one of these two bars was always vertical, and one was always horizontal. Second, we used only two cue colors (red vs. blue). Red cues corresponded to the positive or negative color depending on the search task, while blue cues were non-matching in both tasks. Experiment 3 consisted of 768 trials, with three self-paced breaks after every 192 trials. The procedure of Experiment 3 is illustrated in Figure 5.

#### Participants

Thirty participants (25 females; *Mdn*_Age_ = 21 years, range 18–28 years) took part in Experiment 3, and no participant was excluded as an error rate outlier based on the result of a generalized ESD test.

### Results

We excluded 1.22% of all trials as timeouts and excluded wrong trials (10.91% of all trials). In each cue condition of the negative search task, 40 of 48 (*SD* = 4) valid trials remained on average, and in the positive search task, 43 of 48 (*SD* = 3) valid trials remained per cue condition. All ICC2s were above .76, which was found with blue cues in valid trials of the negative search task.

#### Validity effects in reaction times

We found that a model including random by-participant intercepts and an interaction between the fixed factors cue condition and search task described our data significantly better than a model including only the main effects of our fixed factors, χ^2^(1) = 29.34, *p* < .001. Again, we found that adding a main effect of or interactions with task-repetition did not significantly improve the model fit, χ^2^(1) = 0.05, *p* = .828.

**Fig. 6:**
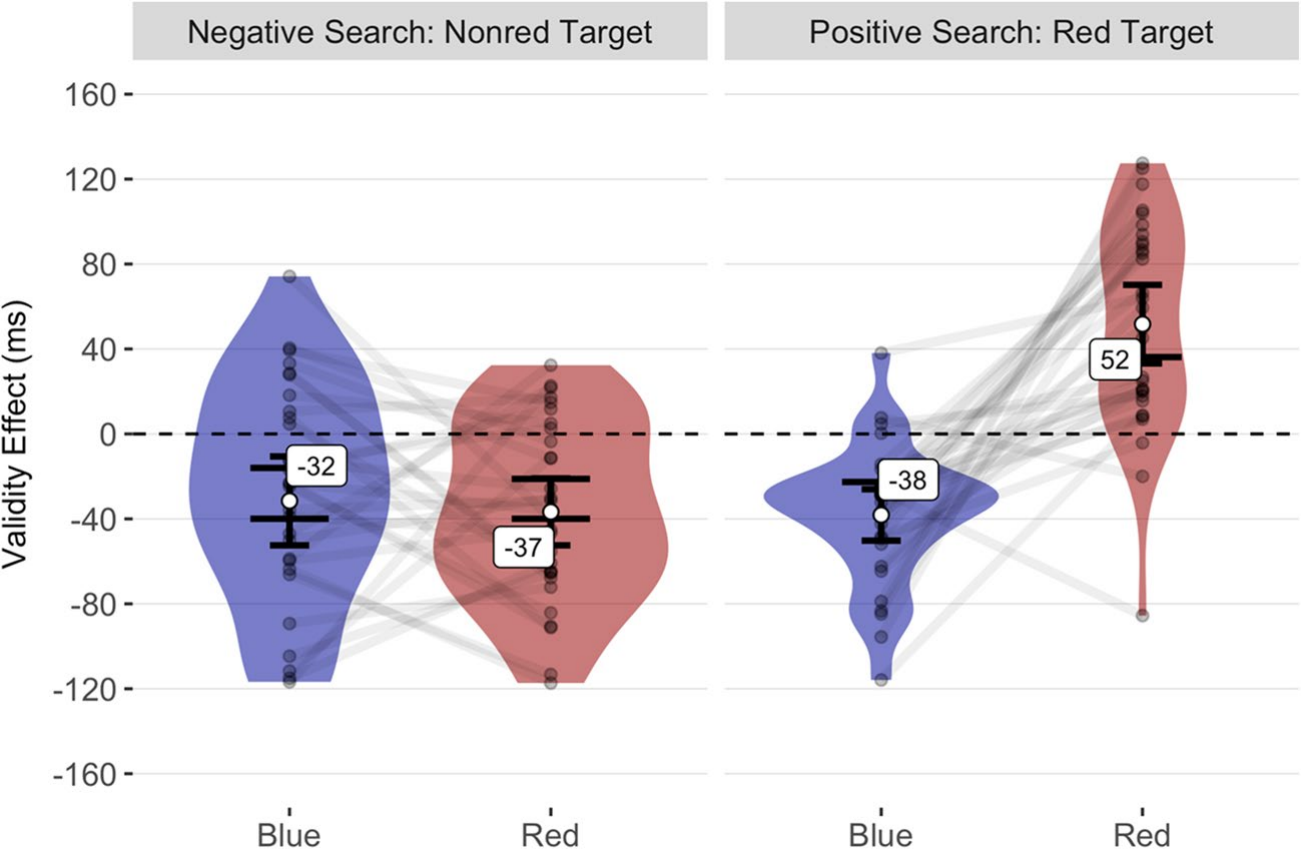
Mean validity effects of Experiment 3. The mean validity effects are shown on the *y* axis as a function of the cue condition and search task on the *x* axis. The short error bars represent the 95% CIs for the one-sample *t* test against zero ms (black dashed line). The validity effect difference between cue conditions is significant if the long error bars do not overlap. The semitransparent points represent the mean individual validity effects, while the violin plots show their distributions. Lines connect the values of each participant across different cue conditions within search tasks

In the positive search task, we found a significant standard validity effect for red cues, *M* = 52 ms, 95% CI [33, 70], *SD* = 50ms, *t*(29) = 5.72, *p* < .001, *d*_unb_ = 1.02 [0.59, 1.48], whereas in the negative search task red cues elicited a significant inverse validity effect, *M* = −37 ms, 95% CI [−52, −21], *SD* = 42 ms, *t*(29) = −4.76, *p* < .001, *d*_unb_ = −0.85 [−1.29, −0.44]. For blue cues, validity effects were similarly inversed in the positive search task, *M* = −38 ms, 95% CI [−50, −26], *SD* = 32ms, *t*(29) = −6.45, *p* < .001, *d*_unb_ = −1.15 [−1.64, −0.7] and the negative search task, *M* = −32 ms, 95% CI [−52, −11], *SD* = 56 ms, *t*(29) = −3.08, *p* = .009, *d*_unb_ = −0.55 [−0.94, −0.17]. Between conditions, we found a significant validity effect difference between red cues in the positive versus the negative search task, Δ −88 ms, 95% CI [−111, −66], *t*(29) = −8.17, *p* < .001, *d*_unb_ = −1.45 [−2.01, −0.96]. In addition, validity effects differed significantly between red and blue cues in the positive search task, Δ 90 ms, 95% CI [65, 115], *t*(29) = 7.34, *p* < .001, *d*_unb_ = 1.31 [0.84, 1.83]. In contrast, no significant validity effect difference was found between red and blue cues in the negative search task, Δ −5 ms, 95% CI [−29, 19], *t*(29) = −0.43, *p* = 1, *d*_unb_ = −0.08 [−0.44, 0.28]. Based on our power simulations, the estimated achieved statistical power to simultaneously detect significant inverse validity effects for red cues in the negative search task and significant standard validity effects for red cues in the positive search task was 97%. The validity effects for Experiment 3 are shown in Fig. 6.

#### Validity effects in accuracies

Hierarchical model comparisons showed no significant effect of cue condition or search task on accuracy, indicating that participants’ responses were similarly accurate across conditions (all *p* values ≥ .148).

### Discussion

In Experiment 3, we used the same color for the negative and positive search tasks, and targets were red horizontal bars (positive search task) or non-red horizontal bars (negative search task). We found that red cues accelerated target detection in valid conditions of the positive task but slowed it in valid conditions of the negative task. This observation suggests that the same color was flexibly up- or down-weighted depending on its functional meaning during the respective search task. However, besides red cues in the negative task, similar inverse validity effects occurred with non-matching blue cues in both tasks. These results indicate that red cues rapidly captured attention or were suppressed in response to the prompt, which informed participants about the upcoming target identity. However, while red cues selectively captured attention in the positive task, suppression occurred in all other used cue conditions.

Similar to Experiment 2, the attentional control setting to suppress red during the negative task seemed to be regularly applied to other colored (blue) cues despite not being instructed. These findings suggest that while searching for targets alternately and unforeseeably defined by a positive or negative feature, negative features guided attention in a less feature-specific way than positive features (see also Forstinger & Ansorge, 2023 for evidence for feature-unspecific inverse validity effects).

However, as outlined in the Discussion of Experiment 2, the generalized suppression of multiple cue colors could reflect an alternative search strategy rather than an inability to apply selective top-down suppression. Specifically, in Experiment 3, participants may have adopted an attentional control setting to search for red in the positive task but suppress red when used as a negative color and other irrelevant colors across search tasks. Such a strategy might have been efficient because all cue colors used were not predictive of target positions and, except for red in the positive search task, were only present with non-targets. Remember that the non-matching cue color differed from any potential target color.

At this point, we do not know why negative and non-matching cues were sometimes similarly suppressed. However, we discuss different possible explanations in the General Discussion. Regardless of this open question, Experiment 3 offers novel insights into the flexibility of top-down attentional control. We showed that the same color can impact attentional guidance differently, through capture or suppression, depending on its functional meaning during visual search. Furthermore, top-down capture or suppression by one and the same feature can be rapidly gated on a trial-to-trial basis, depending on current instructions.

## General discussion

The current study addressed three questions regarding the flexible top-down control of visual attention, primarily focusing on negative features. First, we investigated whether working memory-based attentional control settings facilitate visual processing per se or can gate selective suppression based on task demands (Experiments 1, 2, and 3). Second, we investigated whether proactive suppression and enhancement can be swiftly initiated and deactivated depending on the features’ task relevance and current search goals, as would be predicted based on a working memory account (Experiments 2 and 3). Third, we tested whether even the same task-relevant feature can flexibly guide attention through capture or suppression based on its current meaning during visual search (Experiment 3).

In three experiments, we instructed participants to search for a target defined by different negative colors or a negative versus a positive color, depending on a prompt presented at the beginning of each trial. In Experiment 1, participants searched for targets defined by one of two negative colors (Experiment 1; not red vs. not blue). In contrast, in Experiments 2 and 3, targets were defined by a positive or a negative color that were different (Experiment 2; not red vs. blue) or identical (Experiment 3; not red vs. red). Per experiment, search tasks alternated or repeated randomly from trial to trial, but proactive control was allowed through prompts presented at the start of each trial, informing participants about the upcoming target identity.

In Experiment 1, we observed significant inverse validity effects for negative color cues, whereas validity effects for non-matching color cues did not significantly differ from zero, indicating flexible top-down suppression based on task demands. However, with non-red targets, non-matching green cues also elicited inverse validity effects, although they were not statistically significant. Additionally, the validity effect differences between negative and non-matching cues were not significant, indicating some degree of feature-unspecific color suppression during the search for targets by alternating negative colors.

Experiments 2 and 3 showed that positive and negative features influenced attention through facilitating or suppressing target processing at their position, depending on the current search task. Since search tasks randomly alternated or repeated from trial to trial, our findings suggested that participants were flexible in applying top-down attentional control through capture versus suppression. This assumption is supported by our finding that inverse validity effects for cues with negative or positive features were not restricted to task-repetition trials. Instead, they occurred in response to participants’ current use of a negative search criterion to search for the target and, thus, the current task demand for suppression.

Additionally, in the positive but not the negative search tasks of Experiments 2 and 3, cues with the positive feature elicited attentional capture. This was reflected in standard validity effects, with shorter reaction times in valid than invalid conditions. Thus, the positive color cue only captured attention if used as a task-relevant feature necessary to find the target. This flexibility in changing the attentional control settings is in line with an explanation of attentional control based on working memory representations.

However, Experiments 2 and 3 showed that negative features influenced attention in a less feature-specific way than positive features (see also Forstinger & Ansorge, 2023). Participants suppressed cues with the negative and the non-matching (green in Experiment 2; blue in Experiment 3) color in both search tasks. As explained above, some evidence of feature-unspecific suppression also occurred in Experiment 1. These findings indicate that negative features guided attention less selectively than positive features, replicating previous evidence on generalized top-down suppression by working memory-based negative templates (de Vries et al., 2019; Reeder et al., 2017, 2018). However, previous reports of suppression of negative and non-matching colors in tasks with unforeseeably alternating versus repeating positive and negative features could have also reflected an alternative strategy to search for the target rather than an inability to apply selective top-down suppression, which we discuss below.

### The influence of cues not predictive of the target position on attentional guidance

Before we consider possible explanations for different levels of selectivity in attentional control by positive versus negative features, we want to discuss how top-down control by our negative features might have influenced visual attention.

In the present study, we used the validity effect as our primary measure of attentional guidance. It has been suggested that top-down attentional control is proactively initiated before instead of in reaction to the target onset (Ansorge et al., 2005; Ansorge & Horstmann, 2007; Grubert & Eimer, 2020, 2018). Thus, it makes sense that top-down control already applies proactively to target-preceding singleton cues, even though the cues’ processing itself is not particularly helpful for the search task (Folk et al., 1992). Specifically, a cue feature’s influence on attentional guidance, which is reflected in a significant reaction time difference between valid and invalid trials, is assumed to be a result of proactive processing because (1) the cue-target SOA can be short, leaving little room for reactive processing after cue onset and before target onset, and (2) the cue-target SOA is not decisive for the validity effect difference between matching and non-matching cues, meaning the validity effect difference does not increase with increasing head starts of the cues (relative to the targets), as would be predicted by a reactive control strategy (e.g., Ansorge & Heumann, 2003; Chen & Mordkoff, 2007; Remington et al., 2001; Schoeberl et al., 2019). Furthermore, such proactive top-down control must be differentiated from reactive control, which applies after the cue or target display onset, such as the rapid rejection of known distractor features or deallocation of attention from a cued location after the cue captured attention initially (Moher & Egeth, 2012; Theeuwes et al., 2000).

Following these considerations, our inverse validity effects for negative color cues expand current knowledge on top-down suppression. We showed that top-down suppression operates not only through reactive control by rapidly rejecting known distractor features in the target display (Zhang & Carlisle, 2022), but also through proactive suppression of distractor features (Arita et al., 2012). This assumption is supported by the fact that our cue-target SOA of 60 ms was likely too short for reactive control following the cue and prior to the target (Moher & Egeth, 2012). A similar argument holds with respect to deallocation (Kim & Cave, 1999). Furthermore, our study supports previous evidence for early top-down suppression based on attentional control settings for negative features (Zhang et al., 2020). In sum, our results suggest that proactive top-down suppression is contingent on a currently used negative search criterion and the task relevance of the distractor feature during visual search (see also Kerzel & Huynh Cong, 2022). In this sense, top-down control by negative features seems to apply proactively and flexibly, similar to attentional guidance by positive features.

### Object-file updating as a possible source of inverse validity effects

Theoretically, inverse validity effects for non-matching and negative cues could also reflect other processes than suppression such as the costs associated with integrating the feature change between cue and target into a joint object file. To minimize the possible influence of object-file updating on our findings, we took two measures, using a brief cue exposure of 50 ms (Carmel & Lamy, 2015; but see Schoeberl et al., 2020) and a *masking display* between cueing and target display to incur similar feature changes at all stimulus positions, which is assumed to eliminate a selective object-file updating cost at the locations of non-matching and negative cues. However, we note that these approaches may not have entirely prevented object-file updating because evidence suggests that not being aware of the cue, rather than a brief cue exposure (Lamy et al., 2015), seems to prevent inverse validity effects with non-matching cues (see Schoeberl et al., 2020 for inverse validity effects with non-matching cues and cue exposure of 50 ms). Additionally, achromatic colors, such as our white *masking* disks, may trigger a less substantial object-file update than chromatic colors, such as our cue features (Schoeberl et al., 2020).

Nevertheless, in the present study, we can rule out the possibility of object-file updating as an alternative explanation for inverse validity effects based on an exploratory analysis of trials with negative cues. To be precise, we compared the inverse validity effects of each experiment, with different invalid negative cue conditions – that is, with an invalid negative cue at the position of the negative distractor, to trials with an invalid negative cue at the position of a differently colored non-target. Theoretically, object-file updating costs should be smaller in the former condition because there was no color change at the invalidly cued position, resulting in substantial inverse validity effects. In contrast, in the latter condition, object-file updating would occur in invalid conditions similarly to valid conditions, leading to non-significant validity effects. However, we found no significant difference in validity effects between these conditions,^5^ suggesting that a feature change between the cueing and target display did not incur a significant object-file updating cost. Based on these findings, our inverse validity effects are more likely to reflect participants' attentional control setting for suppression rather than processes that are independent of top-down control.

### Possible explanations for different degrees of feature-specificity in attentional guidance by positive and negative features

Nevertheless, the present findings indicate that attentional control settings were less feature-specific for negative than positive features. This assumption is based on our observation of inverse validity effects for negative and non-matching cues in both tasks of Experiments 2 and 3, while standard validity effects occurred selectively for positive cues in the positive search tasks. Some evidence for this lacking specificity was also found in Experiment 1. Although, we cannot provide a definite answer as to why attentional guidance is more feature-specific and suppression is more feature-unspecific, the factors discussed below possibly contributed to this observation.

### Feature-unspecific suppression might reflect a more efficient search strategy

To start with, participants could have noticed and actively suppressed non-matching cue colors because they were the same throughout our experiments (Kerzel & Barras, 2016). The consistent non-matching cue colors and the requirement of a negative template in some trials may have encouraged participants to use a negative control setting for non-matching cues, too. Such a strategy seems reasonable given that the cues were not predictive of the likely target positions. Furthermore, such a strategy would have been also possible regarding the non-matching green and non-matching red cues in Experiment 1. Additionally, in Experiment 2, we might have unintentionally encouraged participants to suppress all singleton cues, except for the positive color in both search tasks.^6^ For example, we instructed participants not to search for the negative color (red) in the negative search task but also told them that color was generally not helpful in finding the target. As a result, participants might have down-weighted all irrelevant colors instead of only the negative color in the negative search task.

Furthermore, since the positive and negative colors were distinct (blue vs. red) and had to be alternately used as a search criterion, we might have also promoted that participants maintained an active control setting to suppress the negative and any task-irrelevant non-matching color across search tasks. For example, if participants had already started suppressing multiple colors in the negative search task, they may have continued to do so in the positive search task. As explained in the discussion of Experiment 2, a strategy based on one optimally tuned attentional control setting alone does not explain why validity effects for blue cues were found in the positive search task only. However, if participants kept an optimally tuned negative attentional control setting that guided attention away from the red color across negative and positive search tasks, this could have further increased the contrast between the target and non-target colors in the positive trials (Navalpakkam & Itti, 2007). Furthermore, maintaining the same top-down control settings to suppress negative and non-matching colors across search tasks would also have been applicable in Experiment 1 and might explain, for instance, why red cues in the blue negative search task elicited small inverse validity effects, despite being task-irrelevant in these conditions. Following these thoughts, feature-unspecific suppression might not have occurred due to the limited ability of negative features to be flexibly and selectively used for attentional guidance or suppression based on a working memory representation. Instead, our findings from Experiments 1 and 2 might have reflected an alternative search strategy as outlined above.

Notably, such an interpretation of our results would need to be extended in light of Experiment 3, where we used the same task-relevant color as the positive and the negative search criterion. In this situation, optimally tuned suppression or down-weighting of the negative color (i.e., red) in the negative and the positive search task would not have supported but harmed performance. Indeed, our results showed that the task-relevant color (red) was suppressed in the negative search task, but captured attention when it was used as a positive search criterion (in the positive search task). However, although we hypothesized that inverse validity effects would become more selective in the negative search task, non-matching blue cues elicited similar inverse validity effects across search tasks independently of our instructions.

Therefore, our findings in Experiment 3 indicate that some degree of color-unspecific suppression remained while participants alternately searched for the target by a positive or a negative feature. In general, such higher precision of feature-specificity in attentional control by positive versus negative features aligns with previous results indicating that during visual search, participants prefer using positive features and that positive features guide attention more efficiently (Arita et al., 2012; Kerzel & Huynh Cong, 2022; Kugler et al., 2015; Rajsic et al., 2020; Zhang & Carlisle, 2022). Overall, an inherent preference for using positive features to guide attention makes sense because these cues carry information similar to the target and can be used to visually search for the target (M. W. Becker et al., 2015). Furthermore, this difference in informational value between positive and negative features for the target search might also explain why evidence for suppression is mainly restricted to difficult search tasks (Conci et al., 2019) or when using a negative feature is mandatory due to task demands (Forstinger et al., 2022; Kerzel & Huynh Cong, 2022).

Although our search tasks in Experiments 2 and 3 elicited significant feature-unspecific suppression of non-matching and negative colors, likely due to relatively inert control settings, suppression for specific features could be rapidly gated based on task demands in other instances. Specifically, in Experiment 3, red cues were suppressed or captured attention depending on whether red was a negative or a positive color, respectively. This finding occurred independently of trial-by-trial task repetitions, highlighting that top-down attentional control flexibly initiates suppression or capture by task-relevant features in direct response to current instructions. Furthermore, this observation is much better in line with an interpretation of participants’ strategic choice of how to deal with the non-matching irrelevant but consistent colors than with an assumed necessity of feature-unspecific suppression.

### Possible influences of attentional resources on selective guidance

Alternatively, our results could indicate that objectively equally task-relevant features elicit guiding representations with different selectivity based on being accessible or used more or less easily (Rajsic et al., 2020). For example, it has been suggested that attentional priority corresponds to working memory resources allocated to a feature during visual search. These working memory resources seemingly determine the precision (i.e., feature-specificity) of an attentional control setting and, thus, its guiding efficiency (Huynh Cong & Kerzel, 2021; Kerzel & Witzel, 2019). Suppose suppressing a feature requires more resources than actively facilitating its processing. In that case, although our negative feature was necessary to find the target in the negative search task, participants might have had less difficulty implementing a feature-specific strategy in the positive search task even if both strategies were endowed with the same resources (Huynh Cong & Kerzel, 2021). Such an imbalance in efficiency could explain why validity effects were more selective for positive than negative features in Experiments 2 and 3. Furthermore, in Experiment 1, inverse validity effects of negative cues tended to be more feature-specific, with blue as a negative color compared to red. These findings could indicate that, despite being equally accessible and necessary to identify the target, different task-relevant features might not all receive the same amount of attentional resources during visual search (Stanković et al., 2023).

### How attention is guided during visual search depends on a task-relevant feature’s meaning

Our findings extend current knowledge on the interplay between and flexibility of feature up- and down-weighting in the attentional system. Experiment 3 showed that the same feature elicited standard or inverse validity effects depending on the current search task, which randomly alternated or repeated from trial to trial. These results suggest that enhancement and suppression not only co-occur when tuned to different features. Instead, both processes, but only one at a time, can be triggered in response to the same feature if it has different or even opposing functional meanings (i.e., being present or absent in the target; i.e., being a positive vs. negative feature) during visual search.

Based on these observations, different possibilities exist for how different meanings are incorporated in attentional control settings. On the one hand, participants might set up two distinct control settings for the same feature but with different effects on attentional guidance (i.e., one set for suppression vs. another set for capture). Nevertheless, in that case, the task-relevant feature might always partly activate the wrong attentional control setting (e.g., the positive set in the negative search task) due to its feature matching both control settings. On the other hand, participants might initiate one attentional control setting for a task-relevant color that can operate differently depending on task demands. Then, regardless of whether the color is defined as positive or negative during a search trial, the same control setting might be used. However, the feature’s meaning might be integrated as an additional two-level factor (positive vs. negative), triggering different consequences (capture vs. suppression) based on the same matching visual input. Considering these two possibilities, including a task-relevant feature’s meaning into one guiding representation would allow more efficient attentional guidance during a task requiring the flexible up- and down-weighting of the same feature. Notably, this assumption does not require that positive and negative attentional control settings activate the same mechanisms (Reeder et al., 2017). Instead, they may only share a front end but may otherwise or even entirely be implemented through unique and separate processes in the visual system, which define the outcome of top-down attentional control.

In any case, our results suggest a complementation of the top-down contingency principle. Top-down control settings apparently do not only determine input selectivity – that is, specify which feature to use for the guidance of attention. Top-down control settings also determine output selectivity – meaning what functional consequences (capture or suppression) are coupled to the input.

### Which memory systems support top-down attentional control by negative features, and when are they involved?

Evidence suggests that a task-relevant feature is represented in working memory during the early task phases. However, the representation for control settings can be shifted to long- term memory if the target-defining feature remains constant across several consecutive trials (Carlisle et al., 2011; Woodman et al., 2007; for a review, see Woodman et al., 2013). In this context, episodic long-term memory (Giammarco et al., 2016, 2021) or procedural skill memory (Ansorge et al., 2021, 2022; Schmid et al., 2021), which have more (maybe unlimited) storage capacity and can form associations between arbitrary units of information, might take over and play key roles in attentional guidance by constant target features.

In the present experiments, we presented search tasks randomly intermixed to promote working-memory maintenance of task-relevant features. However, our procedure does not preclude a role for other forms of memory. For example, participants might have used their working-memory only for prompt-related selection of memory representations for the control of attention stored elsewhere. Additionally, since prompt-to-task relations were fixed, even the selection of task-specific prompt usage might have been relegated to some less capacity-limited memory system outside visual working-memory.

Future studies could use even more taxing task changes, such as a more arbitrary selection of negative and positive features prior to each trial than the relatively simple choice between only two task options used here.

### Obligatory suppression reconsidered

Before concluding, we would like to address the assumed necessity of suppressing negative features in our negative search task. While it is theoretically possible for participants to translate instructions to suppress a negative feature into searching for remaining positive features, we believe that, in the present study, this alternative strategy is unlikely for three reasons. First, searching for three positive colors, let alone a conjunction between these colors and an additional feature from another dimension, is extremely difficult and cannot be done proactively (Kerzel & Grubert, 2022).

Second, if participants initially searched for the three potential target colors before searching for an additional orientation among them, a non-target would have been selected in two out of three trials. Finally, when we tested this possibility in a previous study using a cue with one of the three potential target colors, we did not find evidence for this alternative strategy in the form of a (diminished, e.g., one-third the size of a regular) standard validity effect of such a target-colored cue. Instead, this cue was ignored, indicated by a non-significant validity effect (Forstinger et al., 2022). Therefore, we are confident that suppression of the negative features was an expedient, if not even obligatory, processing requirement in the negative search task.

Furthermore, this aspect of our task differed from most previous studies, in which a negative feature was helpful for finding the target, but searching for positive features alone would have been sufficient and expedient (but see de Vries et al., 2019). This difference is decisive because we would not expect participants to prioritize an optional strategy to suppress non-targets, and participants would likely not even use such a strategy in many situations. This means that only a task requiring obligatory feature suppression is sensitive enough to exhaustively test the possibility of proactive suppression based on flexible working-memory representations. We, therefore, recommend using tasks such as ours in future studies of this question to protect against possible insensitivities in tests of the power of suppression.

## Conclusion

The present study extends current knowledge on the flexibility of visual attentional suppression. Our findings suggest that attentional control settings can elicit suppression based on flexibly changing search goals. Furthermore, our results qualify and extend previous evidence suggesting that top-down suppression mainly operates through reactive control by rapidly rejecting known distractor or non-target features after initial attentional capture. Instead, we showed that suppression also constitutes a flexible and proactive form of top-down control of visuospatial attention, supporting goal-directed search behavior. In particular, we showed that features could be swiftly deprioritized or prioritized based on task demands changing from trial to trial. Lastly, our results indicate that attentional control settings do not only store more or less precise representations of task-relevant features – that is, they do not only show input specificity. Instead, they also contain relevant information about a feature’s functional meaning during visual search, thus, showing output-specificity, too. Based on this conclusion, the same feature might influence visual attention differently if it has different implications for visual search. Which attentional control setting is ultimately in operation seems to be determined by the feature’s meaning during a current search trial.

## Figures and Tables

**Fig. 7:**
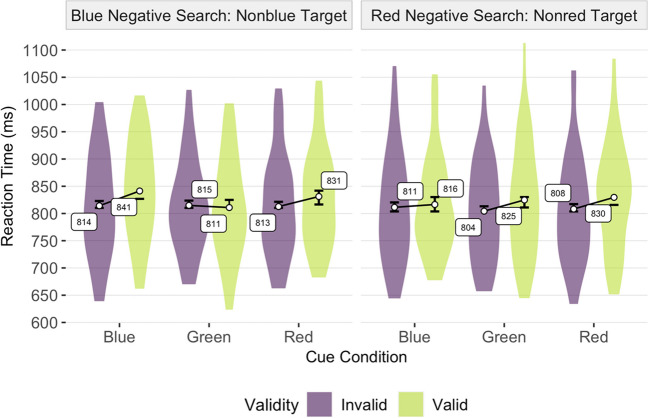
Mean reaction times of Experiment 1. The mean reaction times are shown on the *y* axis as a function of validity (invalid in purple vs. valid in yellow-green), cue condition (blue vs. green vs. red), and search task (blue-negative vs. red-negative) on the *x* axis. The error bars represent the comparison intervals from the R-package *emmeans* for the mean comparisons between all displayed conditions, adjusted with Tukey’s HSD. The largest and smallest values only have one error bar each, since they cannot be compared to a more extreme value. The reaction time difference between conditions is significant if the error bars do not overlap

**Fig. 8:**
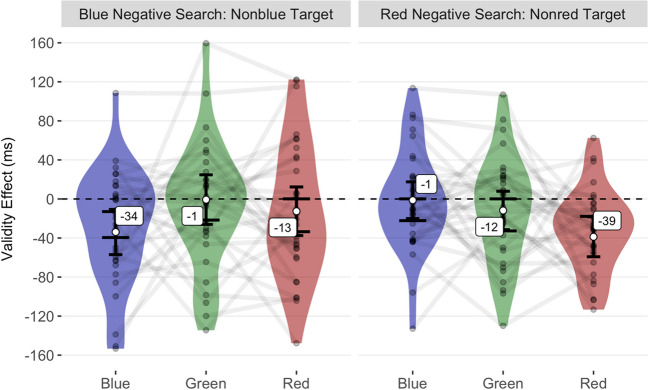
Mean validity effects in reaction times of task-switching trials of Experiment 1. The mean validity effects are shown on the *y* axis as a function of the cue condition and search task on the *x* axis. The short error bars represent the 95% CIs for the one-sample *t* test against zero ms (black dashed line). The validity effect difference between cue conditions is significant if the long error bars do not overlap. The semitransparent points represent the mean individual validity effects, while the violin plots show their distributions. Lines connect the values of each participant across different cue conditions within search tasks.

**Fig. 9:**
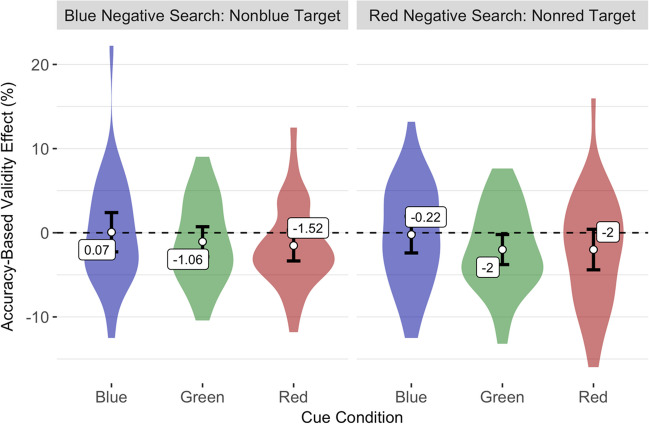
Mean validity effects in accuracies of Experiment 1. The mean validity effects in accuracies are shown on the *y* axis as a function of cue condition (blue vs. green vs. red) and search task (blue-negative vs. red-negative) on the *x* axis. The violin plots show the distributions of the mean individual accuracy-based validity effects. Error bars represent the 95% CIs for the one-sample *t* test against zero ms (black dashed line)

**Fig. 10:**
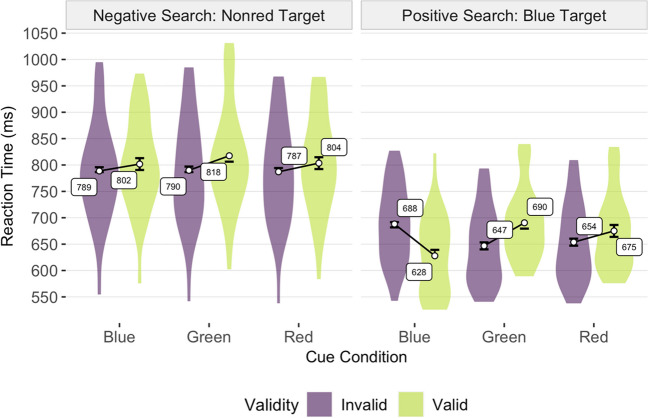
Mean reaction times of Experiment 2. The mean reaction times are shown on the *y* axis as a function of validity (invalid in purple vs. valid in yellow-green), cue condition (blue vs. green vs. red), and search task (negative vs. positive) on the *x* axis. The error bars represent the comparison intervals from the R-package *emmeans* for the mean comparisons between all displayed conditions, adjusted with Tukey’s HSD. The largest and smallest values only have one error bar each, since they cannot be compared to a more extreme value. The reaction time difference between conditions is significant if the error bars do not overlap

**Fig. 11:**
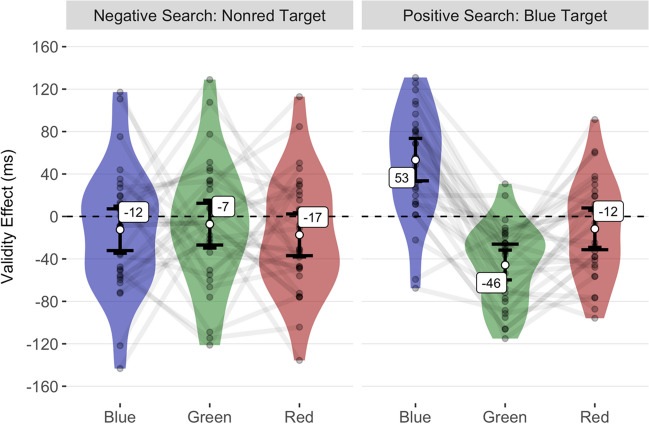
Mean validity effects in reaction times of task-switching trials of Experiment 2. The mean validity effects are shown on the *y* axis as a function of cue condition and search task on the x axis. The short error bars represent the 95% CIs for the one-sample *t* test against zero ms (black dashed line). Between cue conditions, the validity effect difference is significant if the long error bars do not overlap. The semitransparent points represent the mean individual validity effects, while the violin plots show their distributions. Lines connect the values of each participant across different cue conditions within search tasks

**Fig. 12:**
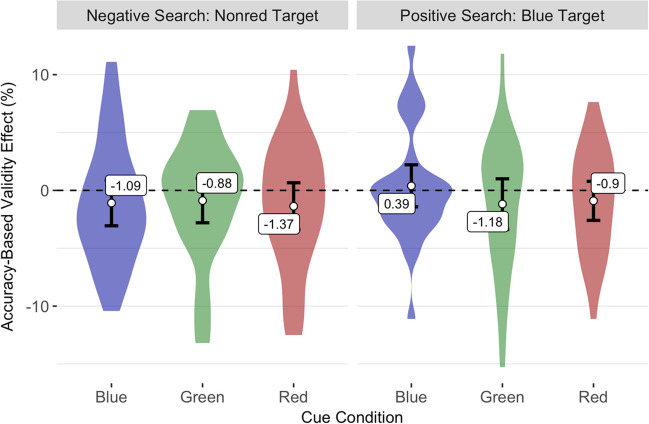
Mean validity effects in accuracies of Experiment 2. The mean validity effects in accuracies are shown on the *y* axis as a function of cue condition (blue vs. green vs. red) and search task (negative vs. positive) on the x axis. The violin plots show the distributions of the mean individual accuracy-based validity effects. Error bars represent the 95% CIs for the one-sample *t* test against zero ms (black dashed line)

**Fig. 13:**
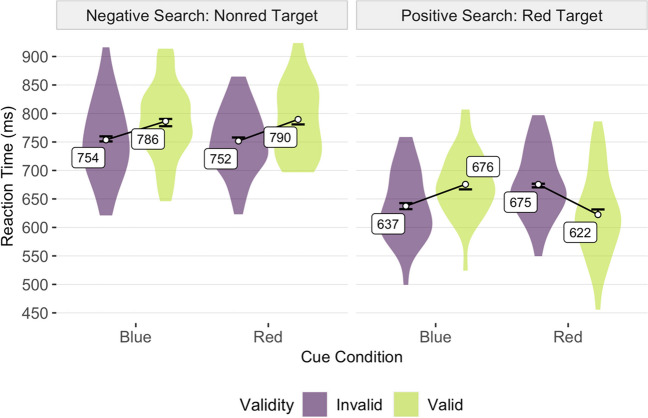
Mean reaction times of Experiment 3. The mean reaction times are shown on the *y* axis as a function of validity (invalid in purple vs. valid in yellow-green), cue condition (blue vs. red), and search task (negative vs. positive) on the *x* axis. The error bars represent the comparison intervals from the R-package *emmeans* for the mean comparisons between all displayed conditions, adjusted with Tukey’s HSD. The largest and smallest values only have one error bar each, since they cannot be compared to a more extreme value. The reaction time difference between conditions is significant if the error bars do not overlap

**Fig. 14:**
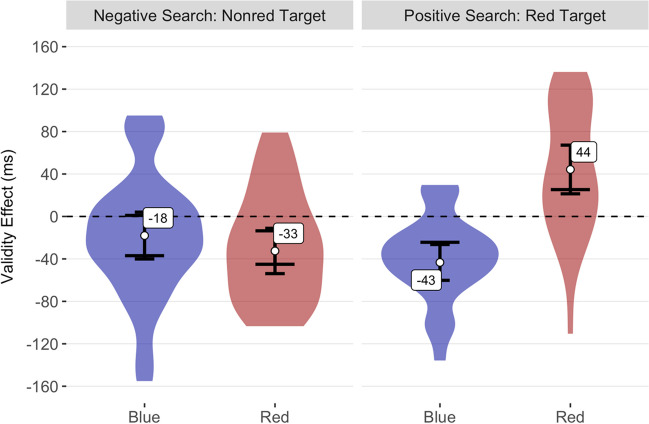
Mean validity effects in reaction times of task-switching trials of Experiment 3. The mean validity effects are shown on the *y* axis as a function of the cue condition and search task on the *x* axis. The short error bars represent the 95% CIs for the one-sample *t* test against zero ms (black dashed line). The validity effect difference between cue conditions is significant if the long error bars do not overlap. The semitransparent points represent the mean individual validity effects, while the violin plots show their distributions. Lines connect the values of each participant across different cue conditions within search tasks

**Fig. 15:**
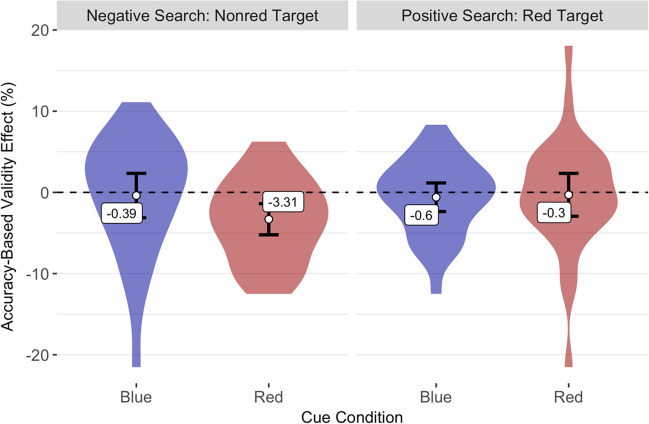
Validity effects in accuracies of Experiment 3. The mean validity effects in accuracies are shown on the *y* axis as a function of cue condition (blue vs. red) and search task (negative vs. positive) on the *x* axis. The violin plots illustrate the distributions of mean individual accuracy-based validity effects. The error bars represent the 95% CIs for the one-sample *t* test against zero ms (black dashed line)

## Data Availability

The raw data of all experiments are publicly available via the Open Science Framework at https://doi.org/10.17605/OSF.IO/8UE3C.

## Appendix

### Mean reaction times of Experiment 1

The mean reaction times for each cue condition and search task are shown in Fig. 7.

### Validity effects in reaction times of task-switching conditions of Experiment 1

The mean validity effects in reaction times of task-switching conditions of Experiment 1 are shown in Fig. 8.

### Validity effects in accuracies of Experiment 1

The mean validity effects in accuracies for Experiment 1 are shown in Fig. 9.

### Mean reaction times of Experiment 2

The mean reaction times for each cue condition and search task of Experiment 2 are shown in Fig. 10.

### Validity effects in reaction times of task-switching conditions of Experiment 2

The mean validity effects in reaction times of task-switching conditions of Experiment 2 are shown in Fig. 11.

### Validity effects in accuracies of Experiment 2

The mean validity effects in accuracies for Experiment 2 are shown in Fig. 12.

### Mean reaction times of Experiment 3

The mean reaction times for each cue condition and search task of Experiment 3 are shown in Fig. 13.

### Validity effects in reaction times of task-switching conditions of Experiment 3

The mean validity effects in reaction times of task-switching conditions of Experiment 3 are shown in Fig. 14.

### Validity effects in accuracies of Experiment 3

The mean validity effects in accuracies for Experiment 3 are shown in Figure 15.

## Footnotes

1 In the current study, we differentiate between reactive versus proactive top-down control for processes that are triggered with or after versus before search display onset (Liesefeld et al., 2023). Nevertheless, we agree with other researchers that both reactive and proactive processes can reflect active top-down control based on participants’ current search goals.

2 While participants may establish attentional control settings for specific features that are currently used as search criteria, it is important to note that these control settings can still be activated by features that differ from the exact feature values of the target. This assumption is based on research showing that when searching for targets defined by specific features, attention is sometimes captured by cues with features similar to the target, suggesting that top-down attentional guidance may not always be as precise (i.e., feature-specific) as it could be (Ansorge & Heumann, 2003; S. I. Becker et al., 2009; for a review, see Yu et al., 2023).

3 We started our hierarchical model comparisons with a null model that included only the random participant intercepts and then added fixed effects one at a time. At each step, we compared the current model to the previous one using a Likelihood Ratio Test to determine if the added fixed effect significantly improved the model fit. We continued this process until adding additional fixed effects no longer resulted in significant improvements in model fit.

4 It must be noted that although we observed inverse validity effects with red cues during the search for non-red targets, these effects were not statistically significantly different from zero. This finding could indicate that participants had difficulties suppressing the negative color while switching between positive and negative colors. However, it is interesting that we found a significant inverse validity effect for red cues while participants searched for blue targets. In Experiment 3, we further investigated how the task demand for switching between a positive and a negative search criterion influenced top-down suppression of the negative feature.

5 For each experiment, we compared validity effects in trials where an invalid negative cue was presented at the negative distractor's position versus a differently colored nontarget's position. However, the results of two-sided paired *t* tests showed no significant validity effects difference between these conditions (all *p* values ≥ 0.228).

6 For blue cues in the negative search task, validity effects were not significantly reversed. Nevertheless, this non-significant result might indicate substantial suppression because blue cues elicited strong standard validity effects in the positive search task. Therefore, rapidly downweighting blue cues in the negative search task, might have resulted in overall neutral validity effects.
